# Novel Compound Heterozygous Pathogenic Mutations of *SLC5A5* in a Chinese Patient With Congenital Hypothyroidism

**DOI:** 10.3389/fendo.2021.620117

**Published:** 2021-03-19

**Authors:** Cao-Xu Zhang, Jun-Xiu Zhang, Liu Yang, Chang-Run Zhang, Feng Cheng, Rui-Jia Zhang, Ya Fang, Zheng Wang, Feng-Yao Wu, Pei-Zhang Li, Jun Liang, Rui Li, Huai-Dong Song

**Affiliations:** ^1^ The Core Laboratory in Medical Center of Clinical Research, Department of Molecular Diagnostics and Endocrinology, Shanghai Ninth People’s Hospital, Shanghai Jiao Tong University School of Medicine, Shanghai, China; ^2^ Department of Endocrinology, Maternal and Child Health Institute of Bozhou, Bozhou, China; ^3^ Department of Laboratory Medicine, Fujian Children’s Hospital, Fujian Provincial Maternity and Children’s Hospital, Fuzhou, China; ^4^ Department of Endocrinology, The Central Hospital of Xuzhou Affiliated to Xuzhou Medical College, Xuzhou, China

**Keywords:** iodine transport, mutation, SLC5A5, next-generation sequencing, congenital hypothyroidism

## Abstract

**Background and Objectives:**

Defects in the human sodium/iodide symporter (*SLC5A5*) gene have been reported to be one of the causes of congenital hypothyroidism (CH). We aimed to identify *SLC5A5* mutations in Chinese patients with CH and to evaluate the function of the mutation.

**Methods:**

Two hundred and seventy-three patients with primary CH were screened for mutations in *SLC5A5* using next-generation sequencing. We investigated the expression and cellular localization of the novel compound heterozygous mutation in *SLC5A5*. The functional activity of the mutants was further examined *in vitro*.

**Results:**

In 273 patients with CH, two previously undescribed pathogenic mutations p.Gly51AlafsTer45 (G51fs) and p.Gly421Arg (G421R) in a compound heterozygous state in *SLC5A5* were identified in a pediatric patient. G51fs was located in the first intercellular loop connecting transmembrane segment I and II, whereas G421R was in the transmembrane segment (TMS) XI. G51fs and G421R resulted in a truncated NIS and reduced protein expression, respectively. *In vitro* experiments further showed that the normal function of iodine transport of sodium-iodide symporter (NIS) mutants was markedly impaired.

**Conclusion:**

The undescribed compound heterozygous mutation of *SLC5A5* was discovered in a Chinese CH patient. The mutation led to significantly reduced NIS expression and impaired iodide transport function accompanied by the impaired location of the NIS on the plasma membrane. Our study thus provides further insights into the roles of *SLC5A5* in CH pathogenesis.

## Introduction

Congenital hypothyroidism (CH), which is defined by inadequate thyroid hormone production in newborn infants, is a common neonatal endocrine disorder with the incidence at about 1:2,000–4,000 worldwide (1). Thyroid hormones are critical for neurodevelopment; severe congenital hypothyroidism can lead to growth retardation and permanent intellectual disability (1, 2).

The human *SLC5A5* (GenBank reference sequence: NM_000453.3) gene, encoding the sodium-iodide symporter (NIS), a 643 amino acid protein, is located on chromosome 19 and consists of 15 exons (3). The NIS protein is composed of 13 transmembrane segments (TMS), an extracellular amino terminus, an intracellular carboxy terminus and three asparagine-linked glycosylation sites. Previous research showed that glycosylation was not critical for NIS targeting to the plasma membrane or normal function (4).

NIS mediates the active transport of iodide from the bloodstream into the tissues, particularly in the thyroid grand, by coupling the inward translocation of Na^+^ down its electrochemical gradient to the inward movement of I^-^ against its electrochemical gradient. The driving force for this process is the electrochemical sodium gradient generated by Na^+^/K^+^ ATPase (2, 5–9). Sufficient dietary iodine intake is essential for the production of the thyroid hormones. In thyroid tissue, iodide uptake is typically the first step in thyroid hormone synthesis. The *SLC5A5* is responsible for the transport of iodide mainly in the thyroid gland (2, 10). The mutation of *SLC5A5* is a cause of thyroid dyshormonogenesis, which leads to CH in patients. Currently, several iodine transport defect-related mutations of *SLC5A5* have been identified, including G18R, V59E, G93R, R124H, Q267E, V270E, C272X, D331N, Y348D, T354P, G395R, R516X, G543E, and S547R, which have provided valuable structural evidence about the symporter and helped us to understand the pathogenesis of this disease (11–24).

The reported mutations of *SLC5A5* almost all affect its normal functional activity, leading to thyroid dyshormonogenesis (5, 10, 25). Our study, therefore, aimed to identify additional mutations in *SLC5A5* from the Chinese patients with CH and analyzed the function of the mutations *in vitro*, to evaluate the roles of *SLC5A5* in the pathogenesis of CH in the Chinese patients.

Herein, we report a novel compound heterozygous missense mutation G51fs/G421R of *SLC5A5* gene in a pediatric patient diagnosed as congenital hypothyroidism from newborn screening. Our results showed that G51fs and G421R markedly affected the function of iodine intake accompanied by impairment of the location of NIS on the plasma membrane. Our study provided further insights into the roles of *SLC5A5* in CH pathogenesis.

## Materials and Methods

### Clinical Subjects

All subjects were recruited from the Chinese Han population in Jiangsu province, Fujian province, Anhui province, and Shanghai through collaboration with multiple hospitals in China. A total of 273 CH patients (141 females and 132 males) identified through newborn screening were enrolled in this study. The diagnosis of permanent CH was confirmed in infants based on the following criteria: CH patients with elevated TSH (thyrotropin) levels, with or without T4 (thyroxine) or FT4 (free thyroxine) levels less than the normal range, and the restoration of normal thyroid parameters after receiving replacement therapy with L-thyroxine; however, after stopping treatment, a rise in TSH and a drop in FT4 were observed again (26–28). Written consent was obtained from the parents of the CH patients, and the study was approved by the Ethics Committee of Shanghai Ninth People’s Hospital affiliated with the Shanghai Jiao Tong University School of Medicine.

### Next-Generation Sequencing (NGS) and Bioinformatics Analysis

The sequencing methods and analysis pipelines used were previously reported (28). Genomic DNA was extracted from the whole blood of the probands and family members if available according to standard extraction procedures. All the exons and exon-intron boundaries of *SLC5A5* were amplified by performing multiplex polymerase chain reaction (PCR) using a 48×48Access Array™ microfluidic platform (Fluidigm) according to the manufacturer’s protocol. The primers were designed using iPLEX Assay Design software (Sequenom). The HiSeq3000 platform (Illumina, San Diego, CA) was used to perform deep sequencing of these amplicon libraries.

The target sequences were amplified and deep sequenced in duplicate for each sample to avoid base pair variants caused by multiplex PCR. Descriptions of the *SLC5A5* mutations were based on NM_000453.3. We analyzed the raw sequence data in fastq format and obtained the quality scores by following the method indicated by previous studies (28). We filtered out the variants with frequencies >1% in the dbSNP 135 and ESP6500 v2 databases and focused on the functional (protein altering) variants (removal of intergenic and 3’/5’UTR (untranslated region) variants, non-splice-related intronic variants, and synonymous variants) identified in duplicate samples (https://doi.org/10.6084/m9.figshare.13134824.v1). Then, the two remaining variants were selected for validation by Sanger sequencing.

### Construction of NIS Plasmids

The ORF (open reading frame, nucleotide 1 to 1932) of human *wild-type* (WT) *SLC5A5* was amplified by PCR from genomic DNA using primers containing the EcoRI and XbaI restriction sites. The amplified PCR fragment was inserted into a p3xFLAG-CMV-10 expression vector and the FLAG tag was inserted upstream to the NIS coding region. Similarly, a WT NIS-EGFP fusion protein was expressed in a pEGFP-N2 expression vector (TransGen Biotech) using EcoRI and BamHI restriction sites, and the GFP was tagged downstream to the NIS coding region. The missense mutations were generated by site-directed mutagenesis using the Fast Mutagenesis System kit (TransGen Biotech). Due to premature termination of NIS, p.Gly51AlafsTer45 (G51fs) containing the EcoRI and BamHI restriction sites was cloned into the pEGFP-N2 vector to generate the fusion plasmid. All the plasmid constructs were verified by Sanger sequencing. The primers used are listed in **Supplemental Table 3**.

### Cell Culture and Transient Transfection

The human embryonic kidney 293T cell line was cultured in Dulbecco’s modified Eagle’s medium (DMEM)/high-glucose medium (Gibco) supplemented with 10% fetal bovine serum (FBS; Sigma Aldrich) at 37 °C in a humidified atmosphere containing 5% CO_2_. Transfections were performed on cells by Lipofectamine 2000 Transfection Reagent (Invitrogen) following the manufacturer’s guides. Cells were plated in a 20 mm glass bottom cell culture dish (NEST) and transiently transfected with 1 µg plasmid DNA to detect the cell localization of the WT or mutants of *SLC5A5*-pEGFP-N2 plasmids. Iodide uptake assays were performed on the 293T cells. The cells were cultured in 12-well plates, transfected with 1 µg WT or mutant 3xFLAG-NIS plasmids (cotransfection with 0.5 µg WT and 0.5 µg mutant plasmids to simulate the heterozygous state), and the treated cells were then kept in a humidified incubator at 37 °C with 5% CO_2_ for 48 h.

### Total RNA Extraction and qPCR (Quantitative PCR)

RNA extraction was performed as described previously (27). RNA was prepared using Trizol reagent (Invitrogen, H10522) and the total RNA (1 μg) was reverse transcribed using the cDNA synthesis kit (Takara). Quantitative PCR (qPCR) was performed using TB Green^®^ Premix Ex Taq™ II (Tli RNaseH Plus), ROX plus (Takara), and the ABI QuantStudio 12K Flex Real-time PCR System (Life Technologies). The primers used are described in **Supplemental Table 3**.

### Iodide Transport Functional Studies

The iodide uptake assay was performed as described previously (29). In brief, 293T cells were seeded in 12-well plates, which contained sterilized cover slips (WHB Biotech), incubated at 37°C for 24 h, and transiently transfected alone or co-transfected with WT or mutant pEGFP-N2 plasmids. 48 h after transfection, the 293T cells were washed once in serum-free DMEM medium and incubated for 1 h in 1 ml serum-free medium containing ^131^I at 5 KBq/ml as the only source of iodide. For the inhibition of NIS-mediated uptake, NaClO_4_ (final concentration 1 mM) was included in parallel incubations (30). The cells were washed briefly in Hank’s Balanced Salt Solution (HBSS) buffer and then incubated with 1 ml HBSS for 5 min. The cells were solubilized by the addition of 1 ml 1 M NaOH, and the radioactivity was measured using a γ counter (GC1200, Anhui, China). DNA was determined by the Qubit Fluorometer using ssDNA Assay Kit (Q10212, Thermofisher). Each value represents the mean ± SD of picomoles ^131^I per microgram of DNA of three independent experiments done in triplicate.

### Western Blot Analysis

Representative Western blot analysis of whole cell lysates from transiently transfected 293T cells was probed with anti-FLAG antibodies (AE063, ABclonal) and anti-GAPDH (AM4300, Thermo Fisher Scientific) antibodies. Proteins were visualized by the Odyssey CLx Infra-Red Imaging system.

### Immunofluorescence Analysis of Protein Localization

The 293T cells were seeded in 12-well plates, which contained sterilized cover slips (WHB Biotech), incubated at 37 °C for 24 h, and transiently transfected with WT or mutant pEGFP-N2 plasmids. One day later, the cells were washed twice with PBS (phosphate buffer saline), fixed with 4% PBS-buffered formaldehyde (PFA) for 30 min at room temperature, washed twice with PBS, and then incubated with fluorescent probe Dil (Beyotime Biotech) targeted to the cell membrane for 5 min at 37°C. After washing with PBS, the nuclei were stained with DAPI (Beyotime Biotech) at room temperature for 5 min. Cover slips were mounted, and the plates were examined using a confocal microscope (Nikon A1 Microsystems).

### Molecular Modeling and Electrostatic Surface

The sequence of *SLC5A5* was analyzed using the SWISS-MODEL (31). The 3D structure of the Vibrio parahaemolyticus sodium/galactose symporter (vSGLT) was chosen as the modeling template. Theoretical modeling of the protein structure was performed using PyMOL 2.4 (https://pymol.org/2/).

### Statistical Analysis

Statistical analysis was performed using Prism 8.0 software (GraphPad Software) from more than three independent experiments. Data were presented as the mean ± SD. Comparisons between two groups were analyzed using unpaired two-tailed Student’s t test (*p < 0.05, **p < 0.01, and ***p < 0.001). Differences were considered statistically significant at values of *p* < 0.05.

## Results

### Patient

In our study, mutations in *SLC5A5* for congenital hypothyroidism were screened for in the cohort of 273 CH patients. Only one patient was found to carry the mutations in *SLC5A5* with no other mutations in the reported candidate genes. The proband was a full-term girl, born from healthy non-consanguineous Chinese parents. Newborn screening found an abnormally high TSH level (specific value unknown). At 18 days after birth, diagnostic confirmation of congenital hypothyroidism was achieved by measuring the serum TSH at >100 uIU/ml (normal range 0.34–5.6 uIU/ml), free T4 0.45 ng/dl (normal range 0.58-1.64ng/dL), and free T3 0.85 pg/ml (free triiodothyronine normal range 2.5–3.9pg/ml) (**Table 1**). Her height was 54.5 cm, and her weight was 3.3 kg.

**Table 1:** Thyroid function tests during follow-up before and after thyroid hormone replacement therapy.

	Before therapy	After therapy			Normal Range
		17 d	4 m	1.25 y	
FT3 (pg/ml)	0.85	3.83	4.56	4.32	2.5–3.9
FT4 (ng/dl)	0.45	1.41	1.30	2.74	0.58–1.64
TSH (uIU/ml)	>100	>100	33.43	0.94	0.34–5.6

At 18 days after birth (before therapy), diagnostic confirmation of congenital hypothyroidism was achieved by measuring the serum TSH, FT3, and FT4. The regular thyroid status was monitored at 17 days, 4 months, and 1.25 years after the initial treatment. Abbreviations: d, day; m, month; y, year; FT3, free triiodothyronine; FT4, free thyroxine; and TSH, thyrotropin.

Thyroid hormone supplementation was started immediately after diagnosis, with a daily dose of 28 μg levothyroxine. However, 17 days after initial treatment, thyroid function was still abnormal (TSH level >100 uIU/ml; FT4:1.41 ng/dl; FT3:3.83 pg/ml) (**Table 1**), and the local pediatrician adjusted the daily dose of levothyroxine to 40 μg. Four months after treatment, her height was 75 cm, and weight was 10 kg. Her thyroid function was slightly improved but still abnormal (TSH:33.43 uIU/ml; FT4:1.3 ng/dL; FT3:4.56 pg/ml) (**Table 1**). At the age of 1 years and 3 months, the proband was recruited to our study. Her height was 81.5 cm, and her weight was 11 kg.

The TSH level returned to the normal range during thyroid hormone replacement therapy (TSH:0.94 uIU/ml; FT4:2.74 ng/dL; FT3:4.32 pg/ml) (**Table 1**). Due to poor compliance, the results of thyroid scintigraphy and the concentration of serum thyroglobulin and autoantibody were unavailable. Normal growth was observed during the first years of life. Of note, the patient’s hypothyroidism did not appear to affect her intellectual development. Thyroid ultrasonography showed a normal size and well-located gland. No other members of the family had a known history of hypothyroidism. The proband’s parents were euthyroid with no autoantibodies and normal thyroid volume by ultrasound.

### Identification of the Novel Compound Heterozygous NIS Mutation

**Figure 1:**
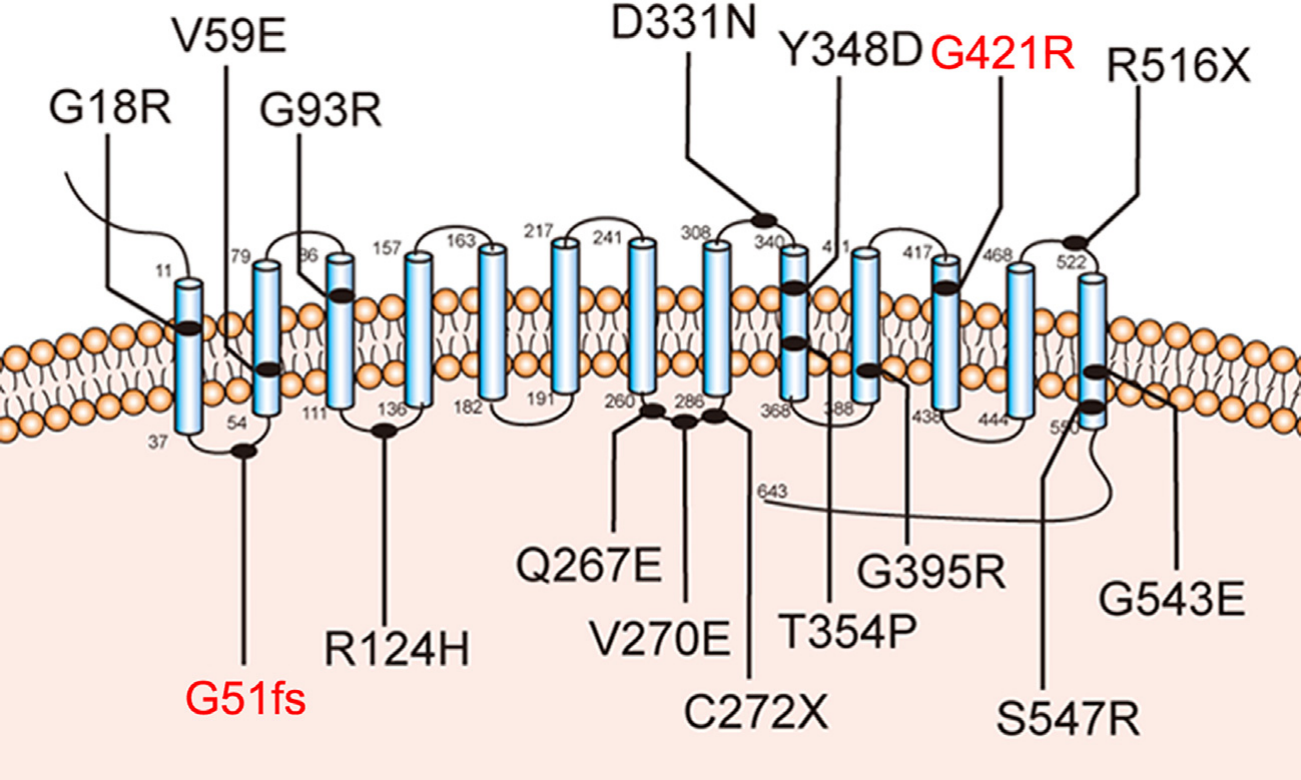
Sodium-iodide symporter (NIS) secondary structure model. Blue cylinders represent the 13 transmembrane segments (TMS). The amino-terminus faces the extracellular space and the carboxy-terminus is in the cytosol. The novel pathogenic variants p.G51fs and p.G421R are highlighted with blue lines.

**Figure 2:**
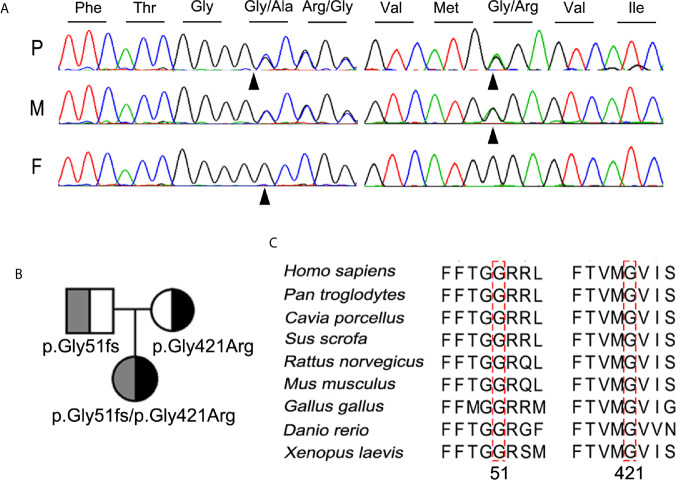
Identification and characterization of the compound heterozygous NIS mutation p.G51fs/p.G421R. **(A)** Partial sequence chromatograms cover the region of the variants in exon 1 (c.152del, p.G51fs) and 11 (c.1261G>A, p.G421R) of *SLC5A5* gene. The nucleotide and corresponding amino acid sequences using the three-letter code are indicated. The proband was compound heterozygous for the mutant alleles p.G51fs and p.G421R NIS. Arrowheads denote the mutated sites. P, proband; F, father; M, mother. **(B)** Pedigree analysis of the members of the proband’s family. The mutated alleles for p.G51fs and p.G421R NIS are noted below. **(C)** Amino acid sequences of NIS from other metazoan species were aligned with those of humans by PSI/TM-Coffee (http://tcoffee.crg.cat/apps/tcoffee/index.html). The mutated amino acids in all NIS homologs are indicated by dotted boxes.

Further molecular analysis of the whole blood of the proband and family members revealed the presence of an undescribed compound heterozygous mutation G51fs/G421R. Based on the current secondary structure model for NIS, G51fs is located in the first intercellular loop connecting transmembrane segment I and II, whereas G421R is in the TMS XI (**Figure 1**). Sequencing of the patient’s *SLC5A5* gene revealed a G deletion at nucleotide +152 in exon 1, resulting in a frameshift mutation and, thus, premature termination of NIS and a loss of about 85% of the amino acids of the protein (**Figure 2A**, **Supplemental Table 1**). The patient also had a novel heterozygous G>A transversion at nucleotide +1261 in exon 11, which leads to the substitution of Gly by Arg at residue 421 (**Figure 2A**).

The mutations contribute to a compound heterozygous mutation G51fs/G421R in NIS (**Figure 2B**). On the other hand, analysis of the euthyroid parents of the proband showed that the father was heterozygous for G51fs NIS, and the mother was heterozygous for G421R NIS, suggesting recessive inheritance of the mutation (**Figures 2A, B**). This information is summarized in a pedigree diagram (**Figure 2B**). Multiple amino acid sequence alignment of NIS orthologs from different species revealed that two glycine residues at position 51 and 421 were highly conserved (**Figure 2C**).

**Figure 3:**
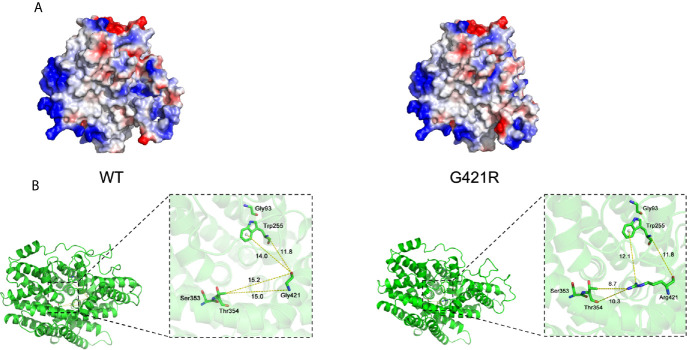
Electrostatic surface and inter-atomic distances of WT NIS and G421R. **(A)** Local solid surface and electrostatic potentials are greatly changed by G421R mutation. Positive and negative potentials are shown in blue and red, respectively. **(B)** The cartoon representation of the crystal structure of NIS based on vSGLT. Those previously reported residues, which are particularly close to the mutation site, are shown in the magnified regions. Dotted lines connect atoms for which inter-atomic distances are given in angstroms. The specific values are shown in the figure. The left part of figure represents WT NIS, and the right G421R.

We next used the Vibrio parahaemolyticus sodium/galactose symporter (vSGLT) as a template to model the 3D structure of G421R. The substitution of the neutral side chain of Gly by the basic amino acid arginine residue at position 421 changed the net surface positive charges and distorted its surface structure (**Figure 3A**). We observed that the interatomic distance between G421 and T354 was changed from 15.0 Å to 8.7 Å (**Figure 3B**). Substituting gly with a bulkier arginine destabilized the helix by causing steric hindrance between the arginine side chain and the nearby amino acid side chains. We further utilized bioinformatic tools to predict the effects of G421R. G421R was categorized by PolyPhen-2 as being “Probably Damaging” with a score of 0.999, and by SIFT as “Damaging” with a score of 0.001 (**Supplemental Table 2**).

### Iodide Transport Activity of G421R and G51fs NIS Mutants

**Figure 4:**
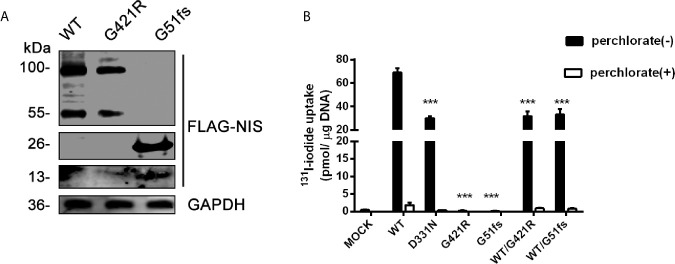
**(A)** Representative Western blot analysis of whole-cell lysates from transiently transfected 293T cells probed with anti-FLAG and GAPDH antibodies. The proteins of WT (wild-type), G421R and G51fs were visualized by Odyssey CLx Infra-Red Imaging system. The molecular weight band was on the right (WT, G421R unglycosylated~ 55 kDa, mature glycosylated~ 100 kDa; G51fs monomer~13 kDa, dimer~ 26 kDa). **(B)** Iodide transport activity for of G421R and G51fs NIS mutants. Mock (p3xFLAG-CMV-10 plasmids), or expressing WT (wild-type) NIS, D331N, G421R and G51fs NIS plasmids were transfected alone or co-transfected into 293T cells. The cells were incubated with with131I at 5 KBq/mL in the absence (black bars) or presence (white bars) of 1 mM perchlorate and the intracellular iodide accumulation was detected using a γ counter. The values are representative of more than three independent experiments and represent the mean ± SD of picomoles ^131^I per microgram of DNA; each experiment was performed in triplicate. ***p < 0.001.

To assess the effect of mutations on the activity of iodide transport, we expressed *wild-type* or mutant 3xFLAG-NIS plasmids in 293T cells. A p3xFLAG-CMV-10 empty vector was employed as a negative control, and a reported D331N mutation construct was used as the positive control. Quantitative PCR analysis was performed to ensure effective transcription (**Supplemental Figure 1A**). Western Blot analysis was done to investigate the effect of G51fs and G421R on protein expression. No band was detected in 293T cells transfected with empty vector (**Supplementary Figure 1B**), while truncated NIS with a molecular weight of 13 kDa (monomer) and 26 kDa (dimer) were observed after G51fs transfection (**Figure 4A**). Moreover, protein expression was decreased in 293T cells transfected with the G421R mutant plasmid compared to those transfected with *wild-type* (immature unglycosylated NIS ~ 55kDa, mature glycosylated NIS~ 100 kDa) (**Figure 4A**).

The iodide transport activity assay demonstrated that 293T cells transfected with WT NIS showed a marked accumulation of radio-iodide in the cells, while addition of the competitive inhibitor perchlorate significantly inhibited NIS-mediated iodide transport (**Figure 4B**). Consistent with the previous study, cells carrying the expression vector encoding D331 NIS exhibited partially reduced, although still detectable, ^131^I uptake, compared with the iodide uptake levels in *wild-type* NIS-expressing cells. G421R and G51fs NIS-expressing cells showed almost no iodide transport activity, with similar radio-iodide levels to cells transfected with a p3xFLAG-CMV-10 empty vector (**Figure 4B**). In addition, substituting of G421 with other amino acids, aspartic acid and phenylalanine), showed the same results (**Supplemental Figure 2**). Further, co-transfection of WT and mutant plasmids to mimic the heterozygous state, no dominant negative effect was observed (**Figure 4B**). Our data showed that G421R and G51fs mutations markedly impaired iodide transport activity of the cells, which was consistent with the impaired membrane patterns.

### Cellular Localization of SLC5A5 Mutants

**Figure 5:**
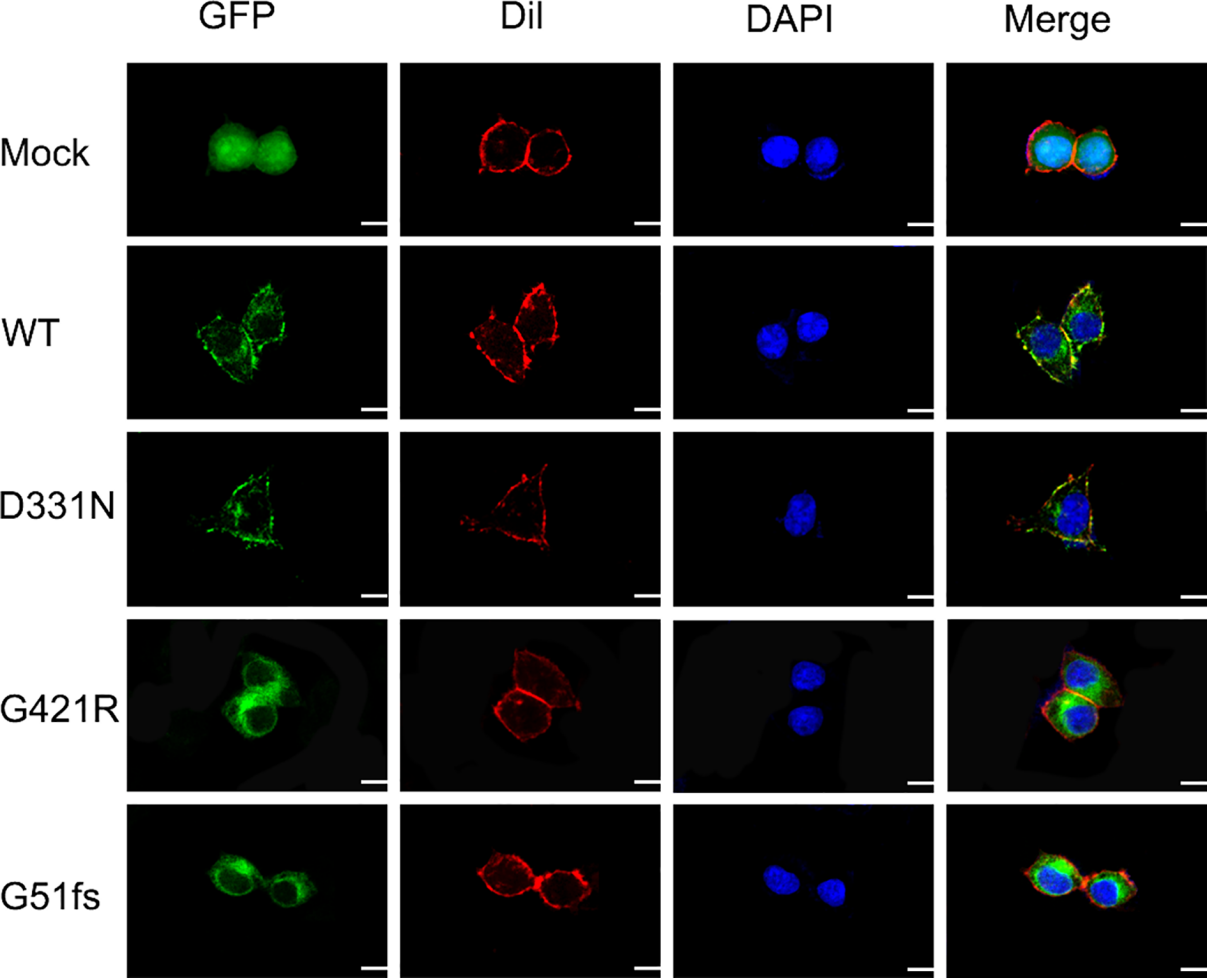
Characterization of cellular localization of G421R and G51fs NIS mutants in 293T cells. pEGFP-N2 empty vector was employed as a negative control (Mock). Human WT NIS, D331N, G421R, and G51fs mutants were cloned into pEGFP-N2 plasmids (green). The cell membrane was labeled with a fluorescent probe Dil (red). Mock, WT NIS, D331N, G421R, and G51fs plasmids were transfected into 293T cells 24 h after seeding. Mutant D331N NIS showed slightly decreased membrane fluorescence, compared with the WT. However, G51fs and G421R mutants displayed severely reduced cell membrane localization in 293T cells. Nuclei were stained with DAPI (blue). Scale: 10 μm.

The sodium/iodide symporter has been shown by immunofluorescence analysis to be located at the basolateral side of thyroid follicular cells and to mediate active iodide trapping. To perform its physiological function, this transporter must be properly localized at the plasma membrane. To assess the effect of *SLC5A5* mutations on the membrane localization, we expressed *wild-type* and G421R or G51fs mutants of the *SLC5A5*-pEGFP-N2 plasmid in 293T cells and examined the cellular localization using a confocal fluorescence microscope. A pEGFP-N2 empty vector was employed as a negative control. As we can see from **Figure 5**, NIS was clearly present at the cell membrane in WT cells, while in D331N transfected cells, normal plasma membrane trafficking of NIS was partially affected. In cells transfected with G51fs and G421R mutants, the NIS expressions on the cell membranes were decreased in varying degrees and appeared to localize in the cytoplasm (**Figure 5**), suggesting impaired plasma membrane localization of the mutated NIS proteins.

## Discussion

In the present study, the undescribed compound heterozygous mutation of G51fs/G421R in *SLC5A5* was discovered in a Chinese pediatric patient with CH. The mutations significantly reduced the NIS expression and impaired iodide transport function accompanied by impairing the location of NIS on the plasma membrane. Our study, thus, provided further insights into the roles of *SLC5A5* in CH pathogenesis.

Clinical heterogeneity in patients with different *SLC5A5* mutations was reported (10). In our patient, hypothyroidism was discovered by neonatal screening. After the diagnosis of CH, the patient was treated with L-thyroxine replacement immediately. However, in many other cases, the onset age of hypothyroidism was variable, ranging from the neonatal period to childhood, and even adulthood (32). Due to delayed treatments, patients presented irreversible intellectual disabilities. Research stated that less than 10% of iodine transport defect patients detected by neonatal screening showed clinical features of congenital hypothyroidism.

In addition, the occurrence of goiters varies between individuals. Because the formation of goiters depends on the level and the duration of TSH overstimulation, as well as on the iodide availability. The patients carrying the same described mutation did not develop goiters. Researchers speculated that early diagnosis and treatment with hormone replacement therapy during the neonatal period might prevent the development of goiters (33). However, even if these patients without goiters at birth are treated and maintain euthyroid, diffuse goiters will still occur after, which indicates that other factors besides TSH stimulation could also cause goiters. Notably, patients carrying the mutation G395R developed goiters has not been reported so far. The majority of these patients were severely hypothyroid, which was detected during the neonatal period (16). The molecular studies of G395R mutation indicated that G395R NIS was synthesized and properly targeted to the plasma membrane but intrinsically inactive (2, 34). It was highly possible that a “milder” pathogenic variant causing a partial loss-of-function might exist and could allow for a lower risk of goiters. Hence, regular long-term follow-up is necessary, and more comprehensive research is required to explore the mechanisms in detail.

In our study, we screened for mutations in *SLC5A5* in a cohort of the 273 CH patients. The frequency of *SLC5A5* mutation in the Chinese patients with CH was about 0.37% (1/273) in our study. However, according to the allele frequency of *SLC5A5* in the Chinese Millionome Database (CMBD), we speculated that the frequency of *SLC5A5* deficiency in CH in Chinese population was about 0.036%, which is significantly lower than what was found in our study. The relatively small cohort size may be a plausible reason for this discrepancy. The G51fs/G421R compound heterozygous mutation in *SLC5A5* in a pediatric patient diagnosed with congenital hypothyroidism has not been reported before. Analysis of the proband’s family showed that the father and mother were heterozygous for G51fs and G421R NIS, respectively (**Figures 2A, B**).

Along with the clinical manifestations of the proband, we proposed that the two novel mutation sites were crucial for normal functional activity of NIS. As expected, cells transfected with the G51fs NIS construct showed no iodide uptake and impaired membrane location due to the loss of most of the functional domains (**Figures 4B**, **5**). Additionally, our studies revealed that the G421R mutation located in the TMS XI was associated with diminished iodide uptake activity (**Figure 4B**). The mutated NIS proteins could not correctly target the plasma membrane (**Figure 5**). *In vitro* experiments in 293T cells transiently co-transfected with WT and mutant plasmids, mimicking the parental heterozygous state, indicated that the mutated allele did not interrupt the activity of WT NIS (**Figure 4B**). This observation was in accordance with the clinical observation that the parents who showed the same mutant allele in the heterozygous state (**Figure 2B**) were euthyroid.

Multiple lines of computational evidence supported a deleterious effect on NIS due to G421R missense mutation (**Supplemental Table 2**). As previously described, the structure of vSGLT, comprised of a central group of seven helices (TMS II-IV, TMS VII-IX, and TMS XI), can form a large cavity participating in the transport of galactose (35). We speculated that the arginine residue may occupy a comparably larger conformational space than glycine in the cavity, thereby, blocking the entry of ions. Previous studies on T354P mutation located in TMS IX revealed that the hydroxyl group at the β-carbon of the residue at position 354 was essential for its function in Na^+^ binding and translocation (23, 24). In addition, the architecture of the Na^+^ binding site was conserved among other transport proteins, such as bacterial leucine transporter(LeuT) and vSGLT (36, 37). We found that the interatomic distance between G421 and T354 was changed from 15.0 Å to 8.7 Å. Replacement of the hydrophobic neutral glycine residue with the hydrophilic and positively charged amino acid changed the net surface positive charges and distorted the structure of the surface, which may disturb the required rigidity of the protein and affect the trafficking of NIS (**Figure 3**). To perform its physiological function of maintaining an active iodide concentration from the blood using the positive sodium gradient, NIS is required to be properly localized at the plasma membrane (30).

In summary, we identified an undescribed compound heterozygous mutation of G51fs/G421R in *SLC5A5* from 273 Chinese patients with CH. These variants were associated with a significant reduction in NIS protein expression and subsequently impaired the membrane localization, resulting in a dramatic reduction in iodide transport and thyroid hormone biosynthesis. Our data demonstrated that these mutations were the direct cause of the observed CH phenotype. This study advanced our understanding of the possible mechanisms of NIS in CH pathogenesis.

## Data Availability Statement

The data presented in the study are deposited in the FigShare repository (https://doi.org/10.6084/m9.figshare.13134824.v1).

## Ethics Statement

The studies involving human participants were reviewed and approved by the Ethics Committee of Shanghai Ninth People’s Hospital affiliated to Shanghai Jiao Tong University School of Medicine. Written informed consent to participate in this study was provided by the participants’ legal guardian/next of kin. Written informed consent was obtained from the individual(s) and minor(s)’ legal guardian/next of kin, for the publication of any potentially identifiable images or data included in this article.

## Author Contributions

C-XZ: writing-original draft and data curation. J-XZ: supervision. LY: project administration. C-RZ: methodology. FC: funding acquisition. R-JZ: resources. YF: resources. ZW: software. F-YW: visualization. P-ZL: validation. JL: funding acquisition. RL: funding acquisition, review, and editing. H-DS: funding acquisition, conceptualization, and investigation. All authors contributed to the article and approved the submitted version.

## Funding

This work was supported by the Chinese National Key Research Program (2017YFC1001801) and the National Natural Science Foundation of China (82070816, 81770786, 81661168016, 81870537, 81670717 and 81870540), and the Shanghai Municipal Education Commission-Gao feng Clinical Medicine Grant Support (20161318).

## Conflict of Interest

The authors declare that the research was conducted in the absence of any commercial or financial relationships that could be construed as a potential conflict of interest.
