# Supplementary material for: Novel Compound Heterozygous Pathogenic Mutations of SLC5A5 in a Chinese Patient With Congenital Hypothyroidism
Source: Front Endocrinol (Lausanne). 2021 Mar 19;12:620117. doi: 10.3389/fendo.2021.620117 (PMC8018529; doi:10.3389/fendo.2021.620117)
Supplement: Supplementary file 1 [file DataSheet_1.docx]

Supplementary Material

## Supplementary Figures

**
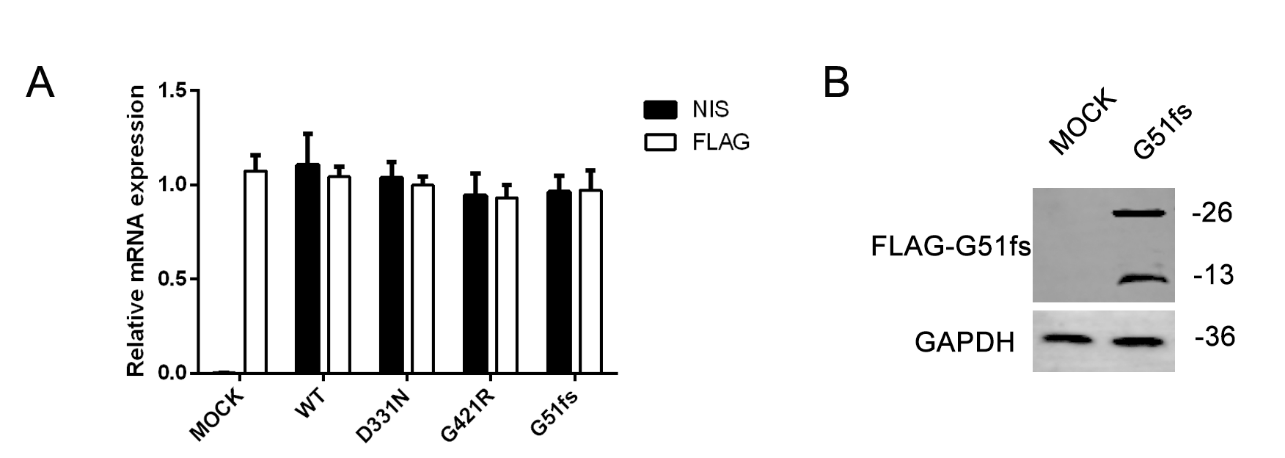
**

**Supplementary Figure 1.** (A) The mRNA level of FLAG and SLC5A5 were measured by qPCR. The results are expressed as the means ± SD. (B) Western blot of 293T cells transfected with the empty vector p3xFLAG-CMV-10 (MOCK) and G51fs expression vector (G51fs).The amino acid alignment of G51fs contained eight cysteine residues. Under the same experimental condition, the presence of disulfide bonds between cysteine residues enhanced protein stability and generated protein dimers. (G51fs ~ 13 kDa, dimer~ 26 kDa.)

**

**

**Supplementary Figure 2.** Steady state ^131^I uptake in empty vector (MOCK) and expressing WT, G421R, G421F or G421D NIS. Cells were incubated with ^131^I in the absence (dark bars) or presence (light bars) of 1mM perchlorate. Replacement with Asp or Phe at residue 421 also led to a markedly decrease in iodide uptake. Results are expressed as means ± SD (pmol ^131^I/μg DNA). Values were representative of ≥ 3 different experiments; in each experiment activity was analyzed in triplicate.

## Supplementary Tables

| MEAVETGERPTFGAWDYGVFALMLLVSTGIGLWVGLARGGQRSAEDFFTGA  GAWRPCPWACRCLPASCRPCRCWACRRRPIAMASSSSGCAWASF* |
| --- |

**Supplementary Table1.** The amino acid alignment of the truncated NIS G51fs.*: termination codon.

| Nucleotide change | Amino acid change | Pathogenicity | ExAC | gnomAD | HGMD | SIFT_  score | SIFT_  pred | Polyphen2_  HDIV_score | Polyphen2_  HDIV_pred | Polyphen2_  HVAR_score | Polyphen2_  HVAR_pred | PROVEAN  score | PROVEAN  pred |
| --- | --- | --- | --- | --- | --- | --- | --- | --- | --- | --- | --- | --- | --- |
| c.152delG | p.Gly51Argfs*45 | P | NA | NA | NA | NA | NA | NA | NA | NA | NA | NA | NA |
| c.G1261A | p.Gly421Arg | P | NA | NA | NA | 0.001 | D | 0.999 | D | 0.99 | D | -7.09 | D |

**Supplementary Table 2.** Results of in silico analysis on the identified variants. SIFT, PolyPhen-2, PROVEAN were used to predict the effects of missense and deletion mutations. P, Pathogenic (pathogenicity rated according to ACMG guidelines); NA, not available; D, Deleterious.

ExAC database (http://exac.broadinstitute.org)

gnomAD database (http://gnomad.broadinstitute.org/)

The Human Gene Mutation Database (HGMD) (<http://www.hgmd.cf.ac.uk>).

| Primers | (5’ to 3’) Fragment |
| --- | --- |
| FLAG-NIS-clone | F: CCGGAATTCATGGAGGCCGTGGAGACCG  R: ACGAGATCTTCAGAGGTTTGTCTCCTGCTGGTCT |
| G50fs-EGPF-clone | F: CCGGAATTCATGGAGGCCGTGGAGACCG  R: CGCGGATCCGAAGCTGGCCCAGGCACATCC |
| D331N- mutagenesis | F: ACATGCCTCTGCTGGTGCTGAACATCTTCGA  R: TCAGCACCAGCAGAGGCATGTACTGGTCTGG |
| G421R- mutagenesis | F: AGGGCTCCTTCACCGTCATGAGAGTCATCAGC  R: TCATGACGGTGAAGGAGCCCTGAAGGACACT |
| G50fs- mutagenesis | F: TGGCGGCCCTGCCCGTGGCCTGTCGCTGT  R: CACGGGCAGGGCCGCCAGGCGCCGGCC |
| NIS-qPCR | F: GCAGTACATTGTAGCCACGAT  R: TGCAGATAATTCCGGTGGACA |
| GAPDH-qPCR | F: ACAACTTTGGTATCGTGGAAGG  R: GCCATCACGCCACAGTTTC |
| FLAG-qPCR | F: GAACCGTCAGAATTAACCATGG  R: CAGATCTATCGATGAATTCGCG |
| G421D- mutagenesis | AGGGC TCCTTCACCG TCATGGACGT CATCAGC  GTCCATGACGGTGAAGGAGCCCTGAAGGACACT |
| G421F- mutagenesis | AGGGC TCCTTCACCG TCATGTTCGT CATCAGC  GAACATGACGGTGAAGGAGCCCTGAAGGACACT |

**Supplemental table 3.** Primer sets used for the construction of plasmids and quantitative real-time PCR. The restriction sites and mutated bases were underlined.


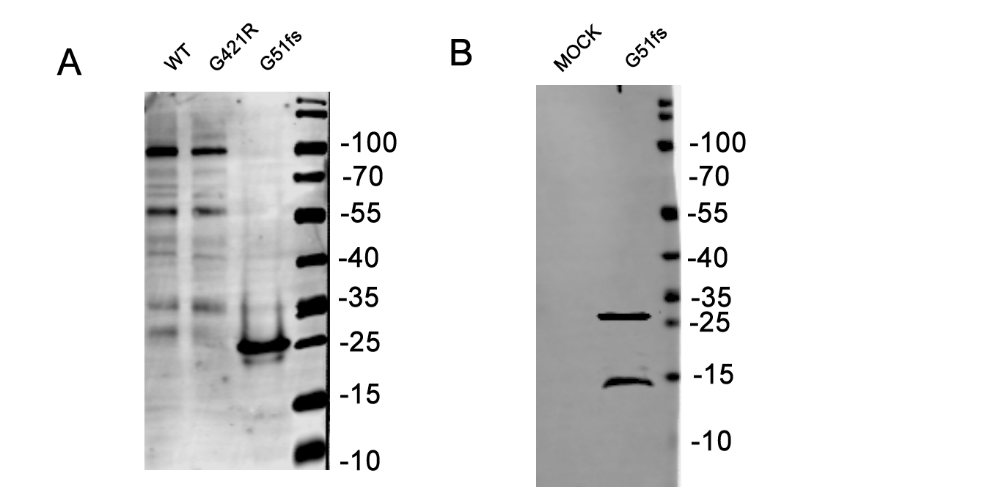


Original images of Western blot based on Odyssey CLx (LICOR). Both glycosylated SLC5A5 (mature form) and unglycosylated (immature form) SLC5A5 were expressed by WT and G421R constructs. (WT, G421R unglycosylated protein ~ 55 kDa, mature glycosylated protein~ 100 kDa; G51fs ~ 13 kDa, dimer~ 26 kDa.)
